# The Effect of the Method of Downhole Deployment on Distributed Acoustic Sensor Measurements: Field Experiments and Numerical Simulations

**DOI:** 10.3390/s23177501

**Published:** 2023-08-29

**Authors:** Boris Gurevich, Konstantin Tertyshnikov, Andrej Bóna, Evgenii Sidenko, Pavel Shashkin, Sinem Yavuz, Roman Pevzner

**Affiliations:** Centre for Exploration Geophysics, Curtin University, Perth, WA 6845, Australia; konstantin.tertyshnikov@curtin.edu.au (K.T.); a.bona@curtin.edu.au (A.B.); evgeny.sidenko@curtin.edu.au (E.S.); pavel.shashkin@curtin.edu.au (P.S.); sinem.yavuz@curtin.edu.au (S.Y.); r.pevzner@curtin.edu.au (R.P.)

**Keywords:** DAS, coupling, numerical simulations, reflectivity method, borehole

## Abstract

Distributed acoustic sensing (DAS) is a promising technology for seismic data acquisition, particularly in downhole applications. However, downhole DAS measurements can be affected by the deployment method of the fibre-optic cable. These effects were explored in a field trial in two wells (one vertical and one deviated) drilled at the Otway International Test Centre. The trial in the *vertical* well shows that (1) fibre-optic cables cemented behind the casing provide data of the highest quality due to the best coupling to the formation, and (2) tubing-conveyed cable shows only slightly weaker coupling, but the data quality can be severely degraded by source-generated noise. A cable loosely suspended in the *deviated* well provided data quality comparable to that of the cemented DAS cable. To better understand the nature of the observed effects, the field experiments were supplemented by numerical modelling with a 1.5D full wave reflectivity algorithm (3D wave propagation in a 1D model), where cement, casing and wellbore were represented by infinite vertical layers. The results show that (1) a cement layer has only a slight effect (<5%) on the DAS amplitude; (2) the vertical strain in a liquid-filled borehole is comparable to that in the formation; and (3) the strain amplitude in the cable is of the same order of magnitude both in the formation and in the fluid. The strain in the cable is zero both when the cable’s Poisson’s ratio is zero and when the borehole fluid is air. The results confirm the feasibility of borehole DAS measurements with fibre-optic cables suspended in a borehole liquid (but not gas!).

## 1. Introduction

Distributed acoustic sensors (DAS) are a new type of seismic receiver that measures temporal variations of strain along an optical fibre [1]. DAS is a fibre-optics sensing technique that creates virtual distributed sensors using optical time-domain reflectometry to detect small changes in the elongation of the fibre [2]. DAS has a broad frequency range from 1 mHz to hundreds of Hz [3,4] and has been employed to record signals from local, regional and distant earthquakes [5]; ambient seismic noise; and borehole-related vibrational signals [6,7]. DAS has been particularly successful in borehole seismic applications (such as vertical seismic profiling or VSP) due to its ability to record seismic wavefields along an entire length of the wellbore [8,9,10,11]. Recent studies show that downhole DAS can have sensitivity and resolution comparable to or superior to downhole geophones [10].

Since DAS measures dynamic strain variations along an optical fibre, these measurements can be affected by the medium in which the fibre is located [12] and the method of its installation [13]. In particular, recent studies show that seismic strain amplitudes recorded by DAS can be used to estimate the elastic properties of the formation: the stiffer the formation, the smaller the strain [12,14]. However, the borehole DAS response, and especially strain amplitudes, can also be affected by the borehole environment [13]. Indeed, the DAS fibre or cable is not located in the formation but can be temporarily deployed as a wireline [15], slick line or any other retrievable cable with metallic or non-metallic armour during a well intervention operation or be placed in the well on a more permanent basis. In the latter case, it can either be cemented behind the casing [10], strapped to the production tubing [11] or even cemented inside the casing string during well abandonment operations [16].

The overall sensitivity and transfer function of the DAS system depends on the fibre and cable properties, deployment method, optical scheme and acquisition parameters. The performance of different optical fibres was addressed in [10,17]. Several studies evaluated different cables, especially in near-surface environments, both experimentally and theoretically [18,19,20,21]. Wuestefeld and Wilks [22] investigated theoretical aspects of the acquisition geometry for downhole data using straight and helically wound cables. Downhole experiments using different cable designs were presented by Correa [20] and Sidenko et al. [23], with the measurements conducted using different cables suspended in the well. Bellefleur et al. [24] compared the performance of straight and helically wound cables cemented in two shallow wells. 

Each deployment method has its own effect on the DAS response through both the differences in the coupling of the sensor and the formation and the presence of a specific noise pattern. Several field studies were conducted to understand these factors. Most of those studies focus on wireline deployment [25,26], while the comparison of several different deployment methods in the same well is rare as it requires several cables deployed in the same well [27].

In Section 2 below, we report the findings of a unique field experiment at the Otway International Test Centre, where several methods of fibre deployment were tested in the same well. In Section 3, these experiments are complemented with numerical simulations. 

## 2. Field Experiment

### 2.1. The Otway Multiwell Monitoring Array

**Figure 1:**
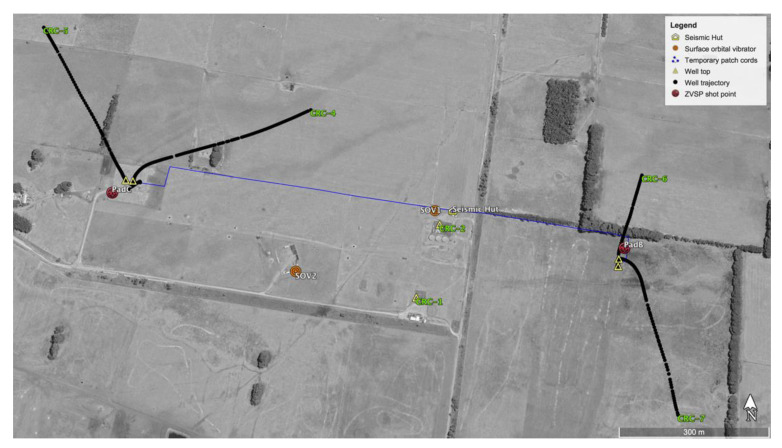
Location scheme. Points marked as PadB, PadC and direct vicinity of SOV1 were used for vibroseis data acquisition.

An array of wells instrumented with fibre-optic cables was built at the Otway International Test Centre (OITC) in the Australian state of Victoria. OITC is a dedicated facility established to conduct field trials of geological carbon storage (GCS) through test injections of carbon dioxide into geological formations and monitoring injected fluids from the surface and boreholes [28,29]. The Stage 3 Otway Project at OITC is focused on the downhole monitoring of a small-scale (15,000 t) CO_2_ injection [30]. The monitoring is performed using an array of several 1.5–1.7 km deep wells drilled within an area of approximately 1 km^2^ [31]. The wells have a combination of several fibre-optic cables deployed using different approaches and, in some cases, have several cables deployed differently in the same well (Figure 1):

- CRC-1 is a vertical well originally drilled for the Stage 1 Otway Project [28]. This well has no permanently deployed fibre optics but can be used to run wireline tools. CRC-2 is another vertical well; it is equipped with a fibre-optic cable with single-mode (SM) and multimode (MM) fibres deployed on production tubing;
- CRC-3 is a vertical well drilled to be the CO_2_ injector for the Stage 3 project. It has two fibre-optic cables cemented behind the casing, one cable having SM and MM fibres while the other also has an engineered single-mode fibre with enhanced backscattering, so-called Constellation Fibre (CF) [10]. One cable is deployed to the total depth while the other is terminated above the perforation interval as a contingency in case of accidental damage to the first cable during the perforation. In 2019, CRC-3 was perforated and completed with an extra fibre-optic cable deployed on production tubing carrying a combination of SM, MM and CF fibres;
- The CRC-4, 5, 6 and 7 wells are deviated monitoring wells with a maximum inclination of 20 to 25 degrees. Similar to CRC-3, each of these wells has two cables cemented behind the casing. However, in these monitoring wells, each cable pair has enhanced backscattering fibres from several different manufacturers. In addition, CRC-4 is also instrumented with a suspended tubing-encapsulated cable with a combination of SM and MM fibres.

The present study analyses the data from the SM fibres only.

The site also has two permanently deployed surface orbital vibrators (SOVs) [20], which were also used as seismic sources in these trials. Locations of the SOVs are marked in Figure 1. Sweep parameters are 0–80 Hz, 155 s and 10 t peak force at 80 Hz. For comparison of the sources, data were also acquired with vibroseis sources located in the direct vicinity of SOV1. 

### 2.2. Field Data Analysis

**Figure 2:**
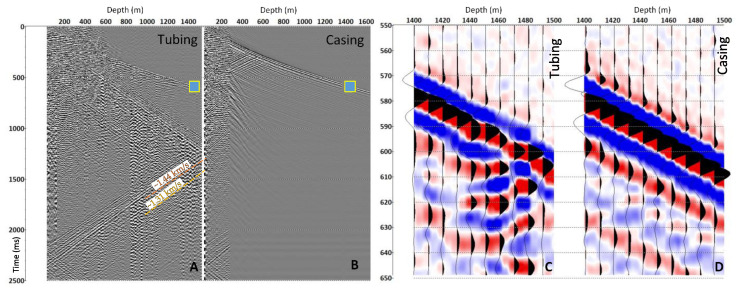
Comparison of DAS VSP in the CRC-3 well acquired using a single vibroseis sweep and recorded by an enhanced backscattering fibre deployed in tubing-conveyed (**A**) and casing-conveyed (**B**) cables. Panels (**C**,**D**) are fragments of the records shown in (**A**,**B**) as blue boxes.

Figure 2 shows a comparison between the zero-offset VSP data acquired in the CRC-3 well using the cables cemented behind the casing and attached to the production tubing; the same amplitude scaling is used in displays A vs. B and C vs. D (which show in colour small fragments of panels A and B, respectively). The largest difference between the two datasets is in the noise field related to waves that propagate along the tubing itself. These waves significantly contaminate the tubing-conveyed cable data (compare A vs. B). Several different tube wave modes with slightly different apparent velocities for the same depth are pronounced on this record (A) (the tube waves’ events and their velocities are highlighted). A tube wave is also present in the cemented cable record but is much weaker (apart from the top 300 m, where the main casing string is not cemented within the surface casing). The cemented cable data previously acquired in the same well before the installation of the production tubing had all the tube waves propagating with the same velocity. 

Tubing reverberation caused by various completion components also affects the data recorded with a tubing-conveyed cable and is not present on the cemented cable. 

Finally, panels C and D show that for different coupling between cables and the formation (through cement vs. through the water), the signals are of the same order of magnitude. The direct P-wave on tubing-conveyed cable is only 30–50% weaker compared to the casing-conveyed cable record. 

**Figure 3:**
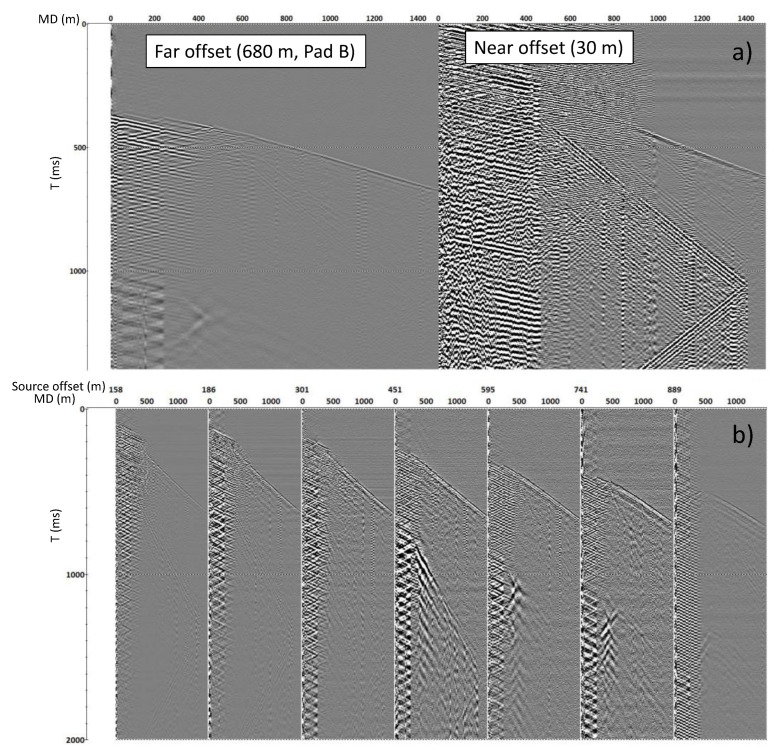
Comparison of DAS VSP in CRC-2 acquired with tubing-conveyed cables using vibroseis at near and far offsets. (**a**) Comparison of seismograms at near (**right**) and far (**left**) offsets—demonstrates the presence and the absence of a tube wave. (**b**) Demonstrates the noise changes with increasing offset.

The tubing-conveyed deployment of an optical fibre for downhole seismic surveys demonstrates the acquisition of high-quality data. A major significant noise component in such installations is tube waves, which are typically generated by energy travelling from the source through the air and impacting the well’s surface infrastructure (wellhead, well tree, etc.). Then, this energy travels along the casing-water/fluid interface, usually with the velocity close to the sound velocity in the borehole fluid, and is sensed by any receivers deployed within the well column. To avoid such prominent noise, the source can be positioned at larger offsets from the wellhead while providing sufficient coverage to obtain an image of the well vicinity. Figure 3 shows examples of data acquired in the CRC-2 well with a tubing-conveyed cable using a vibroseis source at various offsets. The near-offset record (at 30 m in Figure 3a) is heavily contaminated by the tube wave noise, but at a far offset (at 680 m in Figure 3a), it is not present. Figure 3b shows how the noise changes with increasing offset on the CRC-2 tubing-deployed cable. 

**Figure 4:**
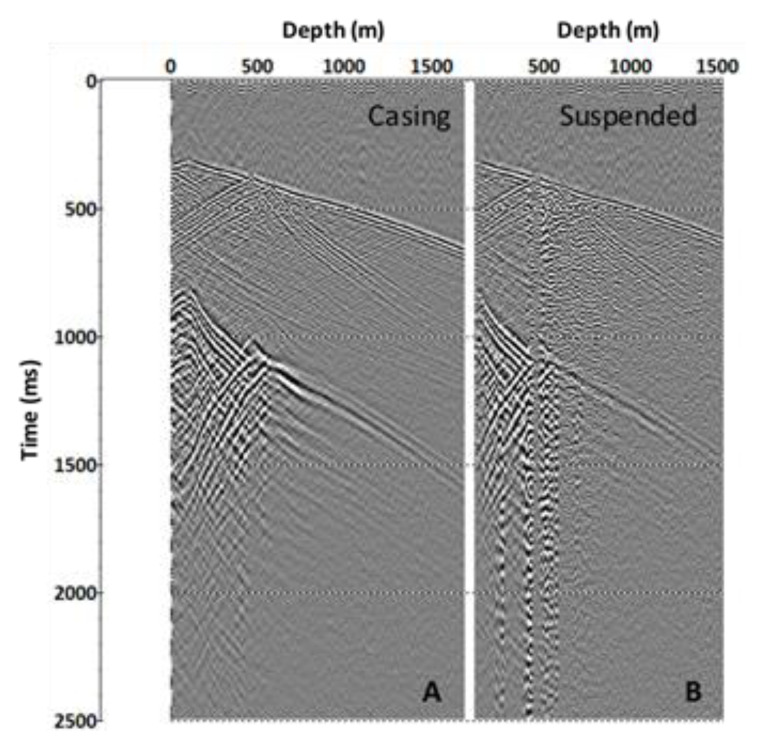
Comparison of DAS VSP in CRC-4 acquired using SOV2 and both cemented (**A**) and loosely suspended cables (**B**), SMF fibre.

A comparison of the performance of the cemented and suspended cables is displayed in Figure 4. In general, the quality of the data acquired with a suspended cable is surprisingly good. The absence of production tubing in CRC-4 reduces the level of source-generated noise, while a slight deviation of the well improves coupling and tension on the cable. This test was carried out using a permanently deployed SOV source after a conventional vibroseis truck had been demobilised from the site.

Some of the results reported in the trial may be surprising, in particular, the excellent results for a cable suspended in the borehole fluid. A quantitative understanding of these effects requires detailed numerical modelling. While adequate numerical modelling of all the 3D effects in the borehole would be cumbersome, as the first step, we conducted simple 1.5D numerical simulations.

## 3. Numerical Modelling

### 3.1. Modelling Approach

We model the effect of the borehole environment with the 1.5D full wave reflectivity method implemented in the OASES software, Version 3.1. OASES simulates 3D wave propagation in a 1D model, which can contain elastic or fluid layers. Similar to the classical reflectivity methods [32], this algorithm involves (1) decomposing the seismic wavefield into plane-wave components, (2) computing reflection and transmission transfer matrices for each component and (3) wavenumber integration to obtain a space–time domain response. The plane-wave transfer matrices are computed using the Direct Global Matrix solution technique, which is much more efficient than classical methods [33]. 

**Figure 5:**
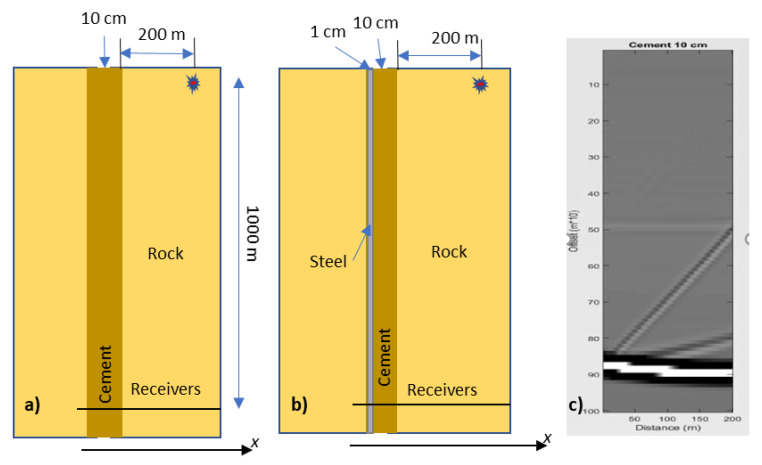
2D layer representation of borehole environments: (**a**) cement layer, (**b**) cement with steel casing; (**c**) snapshot for (**a**) at the time of arrival of the direct P-wave to the receivers.

In our simulations, cement, casing, wellbore and fibre-optic cable are represented by vertical layers of infinite extent (Figure 5a,b). This is done for the simplicity of numerical simulations despite the fact that such a representation severely distorts the axisymmetric shape of these objects and thus can only be assessed qualitatively. The simulations are conducted for a P-wave generated by a point source located 200 m from the borehole wall and 1000 m above the line of receivers, which is located perpendicular to the borehole axis (Figure 5a,b). Of course, in a real borehole experiment, receivers can only be placed in the borehole, but in numerical simulations, they are placed on the line perpendicular to the borehole to explore the variations of strain with the distance from the centre of the well (to see how far the effect of the wellbore propagates into the formation). 

### 3.2. Modelling Results

**Figure 6:**
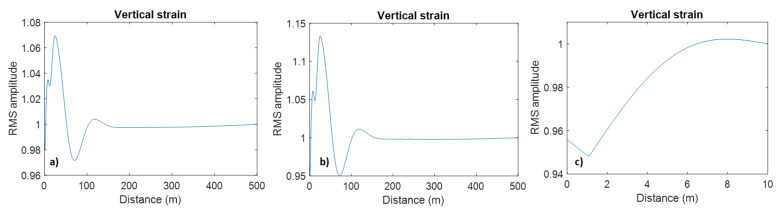
Vertical strain amplitude as a function of lateral coordinate (**a**) for a cement layer, (**b**) for a cement layer with steel casing and (**c**) zoom of (**b**). Please note differences in vertical scale.

**Figure 7:**
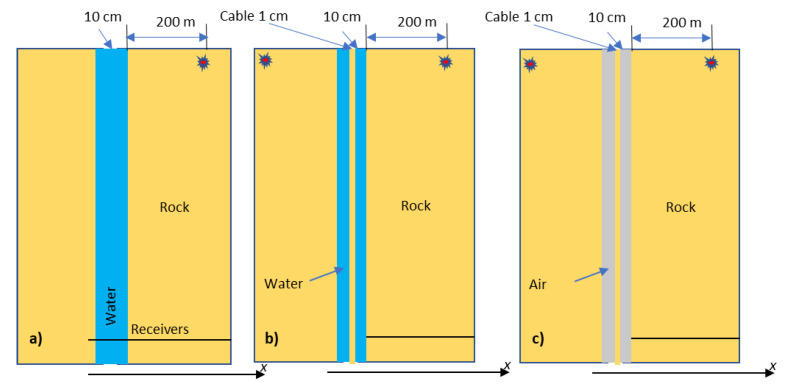
2D layer representation of borehole environments: (**a**) fluid-filled borehole, (**b**) borehole with a cable suspended in water and (**c**) water replaced with air (all modelled as vertical layers).

**Figure 8:**
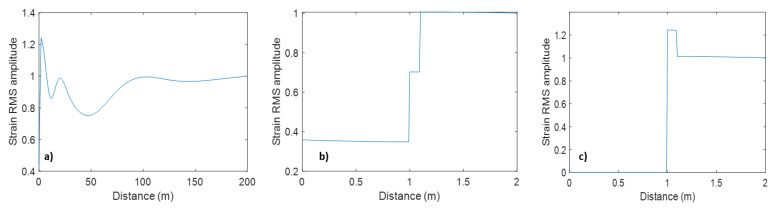
Vertical strain amplitude versus lateral coordinate for (**a**) layer of water (Figure 7a); (**b**) same as in (**a**) but in the vicinity of the layer. (**c**) is the same as in (**b**) but for a layer of air. Note differences in vertical scale.

**Figure 9:**
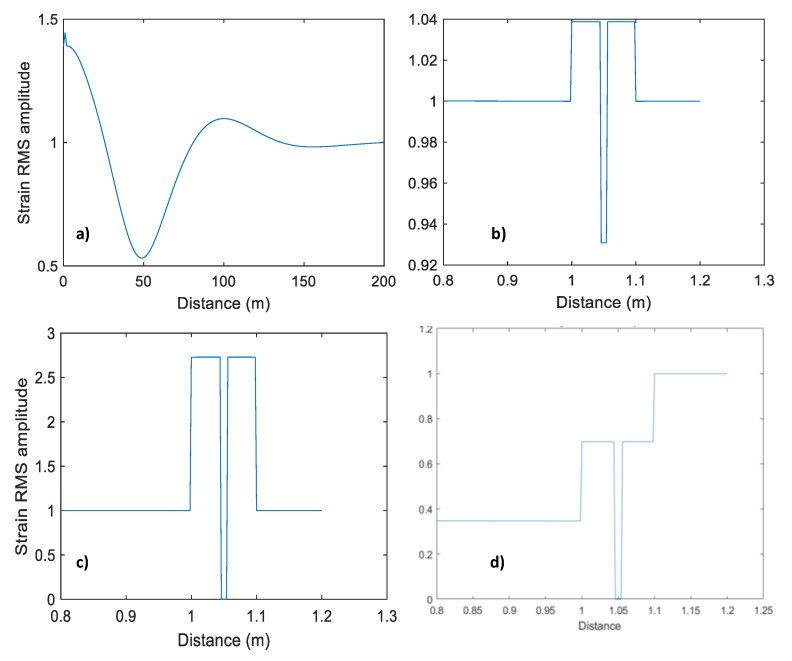
Vertical strain amplitude versus lateral coordinate for (**a**) solid layer (representing a fibre-optic cable) suspended in a layer of water (Figure 7b); (**b**) same as in (**a**) but in the vicinity of the layer. (**c**) is the same as in (**b**) but for water replaced with air (Figure 7c); (**d**) the same as in (**b**) but with the solid layer having zero Poisson’s ratio. Note differences in vertical scale.

**Figure 10:**
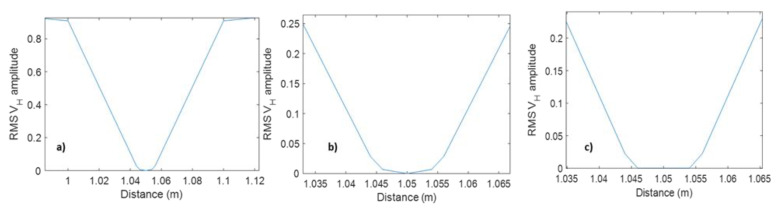
Horizontal particle velocity amplitude versus lateral coordinate for (**a**) solid layer (representing a fibre-optic cable) suspended in a layer of water (Figure 7b); (**b**) same as in (**a**) but within the layer and its immediate vicinity. (**c**) is the same as (**b**) but for water replaced with air (Figure 7c). Note differences in vertical scale.

The modelling results in this section are shown in Figure 6, Figure 7, Figure 8, Figure 9 and Figure 10 as the root-mean-square (RMS) vertical strain amplitude versus horizontal coordinate. For a 10 cm thick cement layer, this strain amplitude in the cement differs from the amplitude away from the well by no more than 2% (Figure 6a). Introducing a 1 cm-thick steel casing increases this effect by a factor of 2, but it is still below 5% (Figure 6b). This small effect of the cement layer on the direct-wave amplitude extends some 100–200 m into the formation (one to two wavelengths). As is evident from the snapshot (Figure 5c), this effect is the result of interference of the direct P-wave with P- and S-wave reflections from the layer.

Figure 8 shows the effect of a liquid-filled borehole represented by a 10 cm thick liquid layer (Figure 7a). The strain in the liquid is of the same order of magnitude as that in the formation but can be larger or smaller, depending on the source configuration (Figure 8a,b). The strain is even larger when water is replaced with air (Figure 8c). 

It should be noted that DAS does not measure strain in the fluid; it measures strain in an optical fibre or cable *immersed in* the fluid. Modelling an optical cable by a 1 cm thick elastic layer (Figure 7b) is shown in Figure 9. This modelling shows that the strain amplitude in this thin solid layer is of the same order but lower than strain both in the formation and in the fluid (Figure 9a, b). However, the strain in the cable vanishes when the borehole fluid is replaced with air (Figure 9c) or when Poisson’s ratio of the cable is zero (Figure 9d). 

Bóna and Lebedev [34] measured the sensitivity of several DAS cables in a water tank to acoustic (pressure) waves in the frequency range from 20 Hz to 1 kHz. They found that the sensitivity of different cables was strongly correlated with Poisson’s ratio of cables’ jackets. These results are broadly consistent with our simulations.

## 4. Discussion

The numerical simulations of the fluid effect on the DAS response are consistent with the physical understanding of the effect of layer properties on wave propagation. Indeed, vertical strain in the ‘cable’ is induced by the horizontal strain through the Poisson-ratio effect. The horizontal strain in the cable is, in turn, induced by the horizontal particle velocity and pressure in the fluid. Thus, for the vertical strain in the cable to be significant, two conditions need to be satisfied:

- The horizontal strain in the cable needs to be significant. The horizontal strain is a lateral derivative of the horizontal particle velocity shown in Figure 10. We see that this derivative is significant when the cable is in water (Figure 10a,b) but zero when it is in air. This is, again, understandable as the transfer of acoustic energy into air is negligible;
- The cable’s Poisson ratio needs to be finite (larger than zero). Materials with a very low Poisson’s ratio (such as quartz glass) are best avoided.

Overall, the results of numerical simulations are consistent with the observations in field experiments. However, it should be noted that the observed effects may be site-specific and can depend on local conditions; therefore, quantitative results cannot be assumed to hold at other sites and will require field experiments at specific sites. In particular, the noise pattern may be different offshore as, here, it is largely generated by surface waves, which are not present in offshore settings. As mentioned earlier, for the same reason, the noise pattern strongly depends on the offset of the source from the wellhead.

All the field experiments in our study were carried out with cables of very similar designs. The use of a cable with a substantially different design may also affect sensitivity. This effect might not be adequately captured by simple elastic modelling presented in our study as cables may contain viscoelastic and plastic components such as a gel.

## 5. Conclusions

The Stage 3 Otway Project provided a rare opportunity to evaluate the performance of the fibre-optic cables for DAS VSP acquisition as a function of the deployment method through direct field experiments. 

As expected, the highest quality of the data was obtained from fibre-optic cables cemented behind the casing. This method provided the best coupling conditions and virtual immunity from tube wave noise. Tubing-conveyed DAS cable demonstrated that coupling of the sensor to the geological formation through the borehole fluid produces a signal-to-noise ratio lower than for the cemented cable but of the same order of magnitude. However, in this case, the data quality can be degraded by the source-generated noise, especially where the tubing is the main conduit. In some cases, this noise can be attenuated by data processing or machine learning techniques.

Cable loosely suspended in a deviated CRC-4 provided data quality quite comparable to the cemented DAS cable. We believe the combination of the deviation of the well and the low tension of the cable positively affected the data quality. 

These field trials were complemented with simple 1.5D numerical simulations. The results confirm the feasibility of borehole DAS measurements with fibre-optic cables suspended in a borehole liquid (but not gas!), provided the cable has a relatively high Poisson’s ratio. However, 2D modelling only provides a very rough proxy for the 3D effects around the borehole. As such, axisymmetric modelling is in order.

The main overall conclusion of this study is that both tubing-conveyed and loosely suspended cables represent viable options for DAS deployment.

## Data Availability

Restrictions apply to the availability of the downhole DAS data. These data were obtained within the CO2CRC Otway project and are available from the authors with the permission of CO2CRC Ltd.
